# Associations Between Maternal Prenatal C-Reactive Protein and Risk Factors for Psychosis in Adolescent Offspring: Findings From the Northern Finland Birth Cohort 1986

**DOI:** 10.1093/schbul/sbaa152

**Published:** 2020-11-17

**Authors:** Hugh Ramsay, Heljä-Marja Surcel, Lassi Björnholm, Martta Kerkelä, Golam M Khandaker, Juha Veijola

**Affiliations:** 1 Department of Psychiatry, Research Unit of Clinical Neuroscience, University of Oulu, Oulu, Finland; 2 Department of Psychiatry, Trinity College, Dublin, Ireland; 3 Faculty of Medicine, University of Oulu, Oulu, Finland; 4 Biobank Borealis of Northern Finland, Oulu University Hospital, Oulu, Finland; 5 Department of Psychiatry, University of Cambridge, Cambridge, UK; 6 Cambridgeshire and Peterborough NHS Foundation Trust, Cambridge, UK; 7 Department of Psychiatry, Oulu University Hospital, Oulu, Finland; 8 Medical Research Center, Oulu University Hospital and University of Oulu, Oulu, Finland

**Keywords:** inflammation, CRP, cannabis, psychotic experiences, birth cohort

## Abstract

Prenatal infection is associated with brain structural and functional abnormalities and may increase the risk for psychosis through a direct effect on neurodevelopment. Various infections may exert their effect through a proinflammatory immune response but studies of prenatal maternal inflammatory markers and offspring neurodevelopment are scarce. Using the longitudinal Northern Finland Birth Cohort 1986 study, we examined the associations of maternal prenatal C-reactive protein (CRP) levels with psychosis risk factors in adolescent offspring. CRP was measured in maternal sera collected in pregnancy. In offspring, school performance was measured at age 7 years, while school performance, psychotic experiences, and cannabis use were measured at age 16 years. We tested associations of CRP with offspring measures using regression analysis controlling for offspring sex, maternal education level, and prenatal maternal body mass index, smoking and alcohol use in pregnancy, place of birth, maternal psychiatric admission, paternal psychiatric admission, mothers age at birth, and gestational week of CRP sample. We also tested if adolescent cannabis use mediated the associations between maternal CRP and offspring outcomes. Controlling for covariates, maternal CRP was associated with academic performance at age 16 years (beta = .062, 95% CI = 0.036–0.088), but not with possible psychotic experiences at 16 years (odds ratio [OR] = 1.09, 95% CI = 0.96–1.24). Maternal CRP was also associated with adolescent cannabis use (OR = 1.24, 95% CI = 1.07–1.43). These findings suggest that prenatal inflammation may influence later mental illness risk by affecting neurodevelopment and also indirectly by increasing the risk of exposure to cannabis.

## Introduction

Evidence from epidemiological birth cohort and other longitudinal studies has linked prenatal infection and risk for psychotic disorders in offspring. Ecological research from Finland,^1^ though not from Holland,^2^ has correlated higher rates of schizophrenia with presence in utero during an influenza epidemic. Further ecological evidence for an association with prenatal infection links the season of birth with schizophrenia.^3^ Importantly, given the methodological shortcomings of ecological studies, epidemiological studies with confirmed prenatal exposure to a range of specific infections during pregnancy have provided support for an association between prenatal infection and offspring schizophrenia.^4,5^ This has included exposure to second-trimester respiratory infections^6^ and to specific infectious agents, such as influenza,^7^*Toxoplasma gondii*,^8^ and herpes simplex virus type 2.^9^ Recent population-based studies from Sweden and Denmark have suggested that the relationship between prenatal infection and offspring psychosis may interact with other factors, including family history^10,11^ and peri-pubertal trauma.^12^

While it is possible that specific infectious agents have individual pathways to illness, one explanation is that there is a common pathway. One possible mechanism is maternal immune activation.^13,14^ Indeed, elevated prenatal inflammatory markers, including C-reactive protein (CRP),^15^ and elevated prenatal cytokines have been associated with offspring schizophrenia.^16,17^

Disruption of fetal development by prenatal maternal infection and immune activation is consistent with the neurodevelopmental model of schizophrenia.^18,19^ Consistent with this idea, maternal infection during pregnancy has been associated with brain structural and functional abnormalities relating to schizophrenia in offspring.^20–24^ Furthermore, studies have suggested that other adverse developmental contexts, such as maternal anemia^25^ and peri-pubertal psychological trauma,^12^ can act along with prenatal infection to further increase the risk.

However, although prenatal infection has been associated with structural and functional abnormalities in the brain, it remains to be seen if it serves as an independent risk factor for psychosis or it exerts its effects on a common pathway with other neurodevelopmental or environmental risk factors. Specifically, there is a notable lack of evidence on the association between prenatal infection/inflammation and early markers and risk factors for psychosis. For example, evidence on adolescent factors such as school performance, psychotic experiences, and cannabis use is limited.

Finnish cohort studies have identified adolescent school performance as a risk factor for later schizophrenia^26^ and adolescent school performance a risk factor for cognitive decline.^27^ Maternal prenatal infection^24^ and maternal elevated cytokines^28^ have been associated with academic ability in early childhood. Evidence is lacking on whether this persists to the key adolescent period. Prenatal infection has also been associated with adolescent psychotic experiences.^29^ However, evidence is lacking on whether prenatal inflammatory markers are also associated with psychotic experiences. Studies utilizing the Northern Finland Birth Cohort 1986 (NFBC 1986) have been able to observe associations between maternal prenatal thyroid function and offspring childhood linguistic and sensory development,^30^ intellectual and scholastic performance,^31^ and inattention and hyperactivity.^32^ This sample, therefore, provides an ideal opportunity to examine the association between maternal immune activation in pregnancy and neurodevelopmental and environmental risk factors for psychotic disorders.

This study aims to examine whether maternal immune activation in pregnancy, measured by circulating CRP (CRP) levels, an archetypal inflammatory marker, is associated with neurodevelopmental and environmental risk factors for psychosis in offspring in adolescence, specifically, psychotic experiences, poorer academic performance, and substance use.

## Methods

### Study Design and Setting

**Fig. 1.**
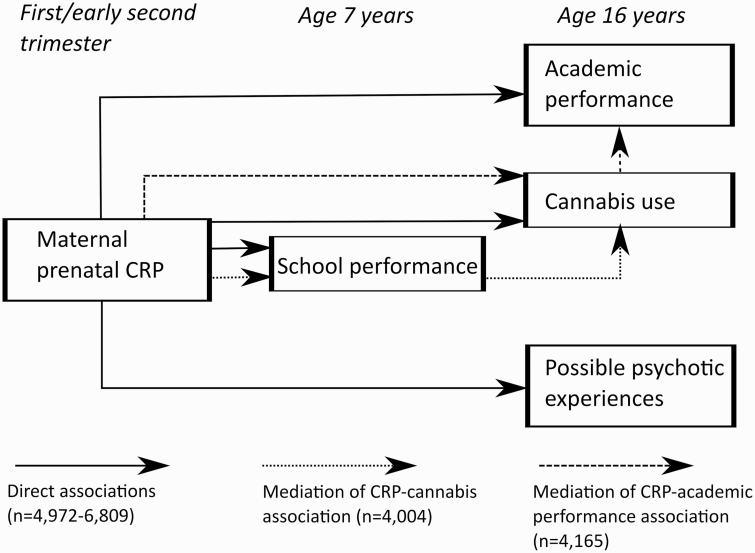
Statistical analyses of direct and indirect associations.

The NFBC 1986 is a longitudinal birth cohort, covering 99% of births in the 2 northernmost provinces of Finland, Oulu and Lapland, who had an expected delivery date between July 1, 1985 and June 30, 1986, details of which have been described elsewhere.^33,34^ In brief, the cohort consisted of 9432 live-born children, of whom 6985 (76%) completed questionnaires^35–39^ at 16-year follow-up in 2001 and 2002 (figure 1).

### Measures

#### Collection of Maternal Sera and CRP Measurement.

Maternal sera were available in the Finnish Maternity Cohort of Northern Finland Biobank Borealis for 7600 mothers of NFBC 1986 members. One serum sample was obtained for each pregnancy during the first or early second trimester of pregnancy. Samples were stored as one aliquot at −25°C in a single, centralized biorepository. High-sensitivity CRP was measured on the clinical chemistry analyzer Architect c8200 (Abbott Laboratories) using a latex immunoassay (Sentinel). During the course of the study, the precision between series expressed as the coefficient of variation (mean ± SD) was 5.1% ± 2.3% and the systematic error (bias) (mean ± SD) was −2.7% ± 7.4. Assay sensitivity was 0.10 mg/dl. The mean CRP value was 4.2 mg/l (standard deviation = 6.4 mg/l). CRP values were not normally distributed and were, therefore, log-transformed and converted to *Z*-scores for ease of interpretation.

Information regarding maternal age and gestational week of blood draw was also collected at sampling. Maternal illicit drug use was also verified using urine drug screen at sampling.

#### Clinical Assessments

Participants were asked about smoking tobacco, alcohol, and drug use at age 16 years. Regarding smoking tobacco, we compared those who had ever smoked tobacco (*n* = 2882, 52.8%) to those who had never smoked it (*n* = 2581, 47.2%). Regarding alcohol, we compared those who had ever been drunk from alcohol (*n* = 3417, 67.3%) to those who had never been drunk from it (*n* = 1657, 32.7%). Regarding cannabis, we compared those who had ever used cannabis (*n* = 297, 5.7%) to those who had never used it (*n* = 4893, 94.3%).

The presence of possible psychotic experiences was judged using the PROD (prodromal symptoms)-screen (screen for prodromal symptoms) questionnaire.^40^We judged the presence of possible psychotic experiences when an individual endorsed possibly hearing voices (item 17), based on evidence that this question is most useful in predicting clinical psychotic experiences.^41^ However, we also required that an individual endorse at least one other “positive” item on the PROD-screen (out of 10 other “positive” items) to increase the specificity of the measure.^42^ In total, 364 out of 4972 (7.3%) scored possible psychotic experiences using this cutoff.

#### Cognitive Assessments

When children were aged 7 years, teachers were asked if the pupil was above average, average, or below average. When adolescents were aged 15–16 years, they completed examinations. Their average performance across academic subjects was converted into a *Z*-score and also divided into 3 equal tertiles. Factor analyses were also performed across performance in history, mathematics, physics, chemistry, biology, geography, art, and music, resulting in 2 factors, one loading for academic subjects and the other for art and music. These factors were also transformed into equal tertiles for analysis.

#### Other Variables.

Information was collected on body mass index (BMI) of the mother during pregnancy and on maternal education level, sex of the child, smoking during pregnancy, alcohol use during pregnancy, and birth area of the child (urban vs rural) during the pregnancy or at birth. For BMI, as described previously,^43^ prepregnancy weight was reported on a questionnaire given to the mothers at their first visit to maternity health centers (on average at the 12th gestational week), while maternal height was measured in 52% of mothers during the same visit and self-reported by the rest.

Information was collected on substance use by questionnaire at age 16 years.

The presence of significant maternal and paternal psychiatric history was assessed using data (up to 2016) from the Finnish Hospital Discharge Register. Parents were classified as having any psychiatric hospital treatment vs no psychiatric hospital treatment.

### Statistical Analysis

Descriptive statistics (*t*-test and univariate regression) were used to characterize the association between maternal CRP levels and demographic factors (sex of offspring, maternal educational achievement, maternal BMI, place of birth [urban vs rural], maternal psychiatric admission, and paternal psychiatric admission), clinical factors (possible psychotic experiences at age 16 years, ever smoked by 16 years, ever used cannabis by 16 years, and ever drunk with alcohol by 16 years), and cognitive factors (teacher’s impression at age 7 years and academic scores at age 16 years). We performed sensitivity analyses by comparing associations with maternal CRP, excluding those with CRP > 10.

Following this, the observed associations between maternal CRP and cannabis use at age 16, school standard at age 7, and total school performance at age 16 were further tested using multivariate regression. These regression models are controlled for offspring sex, maternal education level, maternal BMI during pregnancy, smoking during pregnancy, alcohol use during pregnancy, place of birth (urban vs rural), maternal psychiatric admission, paternal psychiatric admission, mothers age at birth, and gestational week of CRP sample. In comparing univariate and multivariate analyses, only those with data on all covariates were used (unlike in the case of the descriptive statistics above).

Finally, we tested the results for possible mediation. As academic achievement has been associated with later cannabis use,^44^ we examined if the CRP-cannabis association was mediated by school performance at age 7 years. Though debated,^45^ cannabis use may also be associated with poorer academic performance.^46^ We, therefore, examined if the CRP-school performance at age 16 years association may be mediated by cannabis use at 16 years.

Statistical significance was judged significant at *P* < .05 but 95% confidence intervals were also provided for the main outcomes to aid interpretation.

## Results

### Baseline Characteristics of Sample

Maternal prenatal CRP was associated with female sex, poor maternal education, and higher BMI (table 1) and with clinical and cognitive factors (table 2). The mean age of mothers at CRP sampling was 28.2 years (standard deviation = 5.4 years), while the mean gestational week of CRP sampling was 11.1 weeks (standard deviation = 3.6 weeks).

**Table 1.** Maternal CRP Level by Demographic Features^a^

Factor		Mean CRP (SD)	*P*-value for Difference
Sex	Male (3666, 51.4%)	−0.030 (1.003)	—
	Female (3472, 48.6%)	0.017 (0.996)	.009
Maternal education	0–8 years school (624, 10.2%)	0.198 (0.980)	—
	9 and 10 years school (972, 15.8%)	0.066 (0.981)	.010
	Vocational school/college (2788, 45.4%)	−0.039 (1.014)	<.001
	Matriculation (1293, 21.0%)	−0.081 (0.985)	<.001
	Commenced university (468, 7.6%)	−0.057 (0.963)	<.001
Body mass index	Normal weight (5101, 75.4%)	−0.101 (0.977)	—
	Underweight (525, 7.8%)	−0.438 (1.082)	<.001
	Overweight (892, 13.2%)	0.542 (0.785)	<.001
	Obese (244, 3.6%)	0.932 (0.716)	<.001
Place of birth	Rural (1485, 20.6%)	0.06 (0.99)	—
	Urban (5617, 79.4%)	−0.02 (1.00)	.006
Maternal psychiatric admission	No (6538, 91.6%)	−0.001 (1.00)	—
	Yes (600, 8.4%)	0.02 (1.04)	.653
Paternal psychiatric admission	No (6281, 88.0%)	−0.01 (1.00)	—
	Yes (857, 12.0%)	0.08 (1.00)	.009

*Note*: CRP, C-reactive protein.

^a^ *N* includes all those with data available on variable.

**Table 2.** Maternal CRP Level and Later Clinical Difficulties and Cognitive Performance^a^

Factor		Mean CRP *Z*-score (SD)	*P*-value for Difference
Clinical factors			
Possible psychotic experiences age 16	No (4608, 92.7%)	−0.025 (0.992)	—
	Yes (364, 7.3%)	0.096 (0.972)	.025
Ever smoked age 16	No (2882, 52.8%)	0.026 (0.983)	—
	Yes (2581, 47.2%)	−0.038 (0.991)	.017
Ever drunk age 16	No (1657, 32.7%)	0.013 (0.970)	—
	Yes (3417, 67.3%)	−0.029 (1.001)	.153
Ever cannabis age 16	No (4893, 94.3%)	−0.025 (0.992)	—
	Yes (297, 5.7%)	0.152 (0.955)	.003
Cognitive performance			
Standard age 7	Above average (2345, 36.1%)	−0.041 (0.988)	—
	Average (3475, 53.5%)	0.014 (1.004)	.039
	Below average (675, 10.4%)	0.073 (1.011)	.009
Academic age 16	Highest tertile (2145)	−0.084 (0.990)	—
	Middle tertile (2243)	0.000 (0.980)	.005
	Lowest tertile (2421)	0.050 (1.022)	<.001
Academic factor 1	Highest tertile (2193)	−0.014 (0.967)	—
	Middle tertile (2186)	−0.009 (1.005)	.877
	Lowest tertile (2265)	0.006 (1.023)	.517
Academic factor 2	Highest tertile (2216)	0.007 (1.006)	—
	Middle tertile (2230)	−0.027 (0.994)	.255
	Lowest tertile (2198)	0.003 (0.996)	.887

*Note*: CRP, C-reactive protein.

^a^ *N* includes all those with data available on variable.

### Associations of Prenatal CRP With Cognitive Performance at 7 and 16 Years

Before controlling for potential confounders, at age 7 years, teacher-judged average (*P* = 0.039) or below-average (*P* = 0.009) school performance was associated with higher maternal prenatal CRP compared with above-average performance (table 2). However, this association did not persist in sensitivity analyses when CRP values >10 were excluded (change in beta coefficient from .056 with *P* = .004 to .033 with *P* = .112). Furthermore, in multivariate analysis, the observed association did not persist after controlling for sex, maternal education level, maternal BMI during pregnancy, smoking during pregnancy, alcohol use during pregnancy, place of birth (urban vs rural), maternal psychiatric admission, paternal psychiatric admission, mothers age at birth, and gestational week of CRP sample (odds ratio [OR] = 1.02; 95% CI: 0.92–1.12, *P* = .758).

Elevated maternal prenatal CRP was associated with poorer academic performance at age 16 years (*P* = .005 for middle tertile and *P* < .001 for the lowest tertile in comparison with the highest tertile, see table 2). However, no associations were observed between the factor analysis-derived academic factors and maternal prenatal CRP. The association between academic performance at age 16 years and elevated prenatal maternal CRP persisted in sensitivity analyses (change in beta coefficient from .067 with *P* < .001 to .044 with *P* = .005). The association also remained significant after controlling for sex, maternal education level, maternal BMI during pregnancy, smoking during pregnancy, alcohol use during pregnancy, place of birth (urban vs rural), maternal psychiatric admission, paternal psychiatric admission, mothers age at birth, and gestational week of CRP sample (*P* < .001, see table 3).

**Table 3.** Associations Between CRP and Offspring Clinical and Academic Outcomes at Ages 7 and 16 Years

		Unadjusted Analysis		Adjusted Analysis^c^	
Outcome	Sample^a^	Odds Ratio/Beta (95% CI)^b^	*P*-value	Odds Ratio/Beta (95% CI)^b^	*P*-value
Possible psychotic experiences	4089	1.163 (1.03, 1.31)	.014	1.093 (0.96–1.24)	.176
Ever smoked age 16	4596	0.952 (0.90, 1.01)	.097	0.941 (0.88–1.00)	.058
Ever cannabis age 16	4267	1.223 (1.07, 1.40)	.003	1.240 (1.07–1.43)	.003
Standard age 7	5279	1.080 (0.99, 1.18)	.096	1.016 (0.92–1.12)	.758
Better academic performance age 16	5621	0.072 (−0.099, −0.046)	<.001	−0.062 (−0.088, −0.036)	<.001

*Note*: CRP, C-reactive protein.

^a^ *N* includes all those with data available on all covariates (sex, maternal education level, maternal BMI during pregnancy, smoking during pregnancy, alcohol use during pregnancy, place of birth, maternal psychiatric admission, paternal psychiatric admission, mothers age at birth, and gestational week of CRP sample).

^b^Odds ratio for the increased risk associated with 1 standard deviation increase.

^c^After controlling for sex, maternal education level, maternal BMI during pregnancy, smoking during pregnancy, alcohol use during pregnancy, place of birth, maternal psychiatric admission, paternal psychiatric admission, mothers age at birth, and gestational week of CRP sample.

### Association of Prenatal CRP With Substance Use at 16 Years

As outlined in table 2, ever having smoked tobacco by age 16 years was associated with lower maternal prenatal CRP (*P* = .017). This association remained significant in sensitivity analyses after excluding those with a CRP level > 10 (beta = −.074, *P* = .009). However, the association was not significant in multivariate analyses, controlling for sex, maternal education level, maternal BMI during pregnancy, smoking during pregnancy, alcohol use during pregnancy, place of birth (urban vs rural), maternal psychiatric admission, paternal psychiatric admission, mothers age at birth, and gestational week of CRP sample (*P* = .058, see table 3). Ever having been drunk with alcohol was not associated with maternal prenatal CRP (*P* = .153, see table 2).

Cannabis use at age 16 years showed a univariate association with elevated maternal prenatal CRP (beta = .177, *P* = .003, see also table 2), which remained significant following sensitivity analyses including only those with CRP < 10 (beta = .185, *P* = .003). The association also remained significant after controlling for sex, maternal education level, maternal BMI during pregnancy, smoking during pregnancy, alcohol use during pregnancy, place of birth (urban vs rural), maternal psychiatric admission, paternal psychiatric admission, mothers age at birth, and gestational week of CRP sample (OR = 1.24, *P* = 0.003).

### Association of Prenatal CRP With Possible Psychotic Experiences at Age 16

Elevated prenatal maternal CRP was associated with possible psychotic experiences at age 16 years (*P* = .025, see table 2). This remained significant (beta = .118, *P* = .039) in sensitivity analyses where those with CRP > 10 were excluded. However, the association was no longer evident after controlling for sex, maternal education level, maternal BMI during pregnancy, smoking and alcohol use during pregnancy, place of birth (urban vs rural), maternal psychiatric admission, paternal psychiatric admission, mothers age at birth, and gestational week of CRP sample (unadjusted OR = 1.16, 95% CI: 1.03–1.31, *P* = .014; adjusted OR = 1.09; 95% CI: 0.96–1.24, *P* = .176, see table 3).

### Results for Mediation Analyses

There was minimal evidence that the association between prenatal maternal CRP and cannabis use at 16 years was mediated by school performance at age 7 years (OR changed from 1.24, *P* = .003 to 1.20, *P* = .014). The Sobel test found no indirect effect (coefficient < .001, *P* = .362) in the context of a low direct effect (coefficient = .005, *P* = .246) and suggested that only 1.9% of the observed (low) effect was mediated by school performance at 7 years.

There was some evidence for mediation of the association between prenatal maternal CRP and school performance at 16 years by cannabis use (beta changed from −.062, *P* < .001 to −.047, *P* = .001). The Sobel test observed an indirect effect of −.005 (*P* = .007) in the context of a direct effect of −.011 (*P* < .005), suggesting that 5.8% of the observed effect was mediated by cannabis.

## Discussion

This study has found that a marker of maternal immune activation in pregnancy is associated with adolescent neurodevelopmental and behavioral risk factors for psychosis, including cannabis use and academic performance, even after controlling for important covariates.

Prenatal maternal infection has been clearly associated with risk for schizophrenia in offspring.^4,5^ It has also been associated with functional and structural abnormalities relating to schizophrenia in offspring.^4,21,22^ However, to date, there has been minimal evidence on a possible link between prenatal immune activation and adolescent risk factors for schizophrenia, such as adolescent academic performance, psychotic experiences, and cannabis use.

### Maternal Immune Activation and Child and Adolescent Outcomes

The results here suggest that maternal immune activation during pregnancy is associated with neurodevelopmental and behavioral features, including poorer academic attainment and increased cannabis use. This is consistent with findings from animal studies that prenatal immune activation results in changes in behavior and central nervous system structure and function.^47^As outlined above, one possible biological mechanism is the effect of proinflammatory cytokines.^16,17^ Systemic inflammatory cytokines communicate with the brain.^48^ Maternal immune activation and resultant cytokines can impact on neurogenesis, migration, differentiation, and apoptosis, leading to diverse adverse outcomes.^49^

Though markers of maternal immune activation have been previously associated with psychotic disorders,^15^ it is unsurprising that the univariate association of a marker of maternal inflammation and possible psychotic experiences did not survive controlling for key covariates. Maternal immune activation results in heterogeneous outcomes for offspring, with varying levels of resilience and susceptibility to a wide range of outcomes.^50^ The findings here must be viewed in this context with other prenatal and childhood factors likely interacting with the timing and intensity of maternal immune activation in pregnancy in its association with adolescent outcomes. In the case of possible psychotic experiences, controlling for a number of these prenatal factors (eg, maternal BMI, maternal substance use, and parental psychiatric history) eliminated any evidence for an association. Additionally, the observed maternal CRP levels in those who later have possible psychotic experiences were only marginally higher than in those who did not have these experiences. This may reflect that adolescent psychotic experiences are relatively common^51^ and that only a minority of adolescents who describe psychotic experiences later develop a psychotic disorder.^52^ Psychotic experiences in adolescence also appear to be a heterogeneous group, including many who develop nonpsychotic disorders or no disorders at all.^53^ Psychotic experiences are a relatively “softer” neurodevelopmental feature compared with psychotic disorders.

The finding of an association between maternal immune activation and adolescent cannabis use is perhaps the most surprising. It adds to existing evidence that cannabis use and predisposition to psychosis have a shared genetic and environmental basis.^54^ The mechanism of the association between maternal immune activation and adolescent cannabis use may involve circuitry involved in specific cognitive functions, particularly as our study suggests that the impact was not mediated through an effect on broader cognitive function (observed in academic performance). Spann et al found that maternal immune activation during the third trimester was associated with neonatal functional connectivity of the salience network in fetal and toddler behavior.^55^ Increased markers of maternal immune activation have been associated with lower impulse control and associated brain structural findings at 24 months of age.^56,57^ Maternal immune activation has also been associated with poorer working memory at 2 years.^58^ This increased impulsivity and problems with working memory may persist to adolescence, increasing the risk for cannabis use in this group exposed to maternal immune activation. Maternal immune activation in pregnancy has also been associated with dopaminergic maldevelopment in animal models, leading to functional abnormalities implicated in schizophrenia.^59,60^ Given the role of the dopaminergic neurons in the reward system,^61^ this may also be a mechanism through which maternal immune activation impacts offspring cannabis use.

However, there are likely to be a variety of further resilience and susceptibility factors that impact on the pathway from maternal immune activation to offspring outcomes.^50^ Interestingly, controlling for a number of prenatal susceptibility factors in this study did not significantly attenuate the association between prenatal maternal immune activation and offspring cannabis use. However, a significant number of child factors may also play a role. Indeed, a range of other social and environmental factors are likely to play a more significant role in adolescent cannabis use than prenatal maternal immune activation.

This study was not able to examine if cannabis use is a mediator of any association between maternal immune activation and offspring psychotic disorders, due to lack of association between prenatal CRP and offspring PEs in our sample. Nevertheless, the findings reported here call for further investigation into potential mechanisms of association between prenatal CRP and adolescent cannabis use. The effect of inflammation on impulsivity and cognition could be relevant. Animal studies suggest that adolescent cannabis use may interact with maternal immune activation in a number of ways. Adolescent cannabinoid exposure may act synergistically with maternal immune activation in negatively altering the serotonin system.^62^ However, disrupted dopamine function induced by maternal immune activation may actually be attenuated by Δ ^9^-tetrahydrocannabinol.^63^

The results of this study also give clues regarding the mechanism of the maternal inflammation—psychosis association. Maternal immune activation is associated with a range of adverse neurodevelopmental outcomes. It is likely that much of the risk arises due to effects on cognitive factors associated with psychosis. This would include those discussed above: salience networks,^55^ impulse control^56,57^ and working memory,^58^ and reward pathways. In addition, there is a need for further research to examine if the association with adolescent cannabis use has an impact on later risk for psychosis.

### Strengths and Limitations

This study has a number of particular strengths. Firstly, the data were collected from the NFBC 1986. Data collection from the NFBC 1986 commenced before birth and has continued longitudinally to the present day. The rate of follow-up on the cohort was good with over 4000 individuals available. This strengthens the significance of the observed associations between maternal immune activation and the neurodevelopmental and behavioral outcomes studied. Secondly, the study controlled for a range of important potential covariates, including sex of the offspring, maternal education, tobacco and alcohol use during pregnancy, maternal BMI during pregnancy, place of birth, maternal psychiatric admission, paternal psychiatric admission, mothers age at birth, and gestational week of CRP sample. Self-reported illicit substance use in pregnancy was further validated using urine drug screening.

The study would have benefitted from a broader panel of markers of maternal immune activation (eg, interleukin-6 levels during pregnancy) and from maternal illness history factors. However, CRP is a good general measure of immune activation, especially considering we controlled for important factors that can influence this (eg, BMI). Future studies could add to this work by examining other markers of immune activation. A second limitation is that many of the adolescent measures are self-reported (particularly cannabis use and possible psychotic experiences). Ideally, these would have been measured through laboratory study (cannabis use) and clinical interview (possible psychotic experiences). Given the numbers included in the study, unfortunately, self-report was only possible. While self-report of psychotic experiences is a significant limitation, the item was strengthened by requiring both that the person reported the hearing voices item on the PROD-screen and a further additional “positive” score item. Self-reported cannabis use was also low (5.7%) in this study. This likely reflects cultural factors with markedly higher levels of alcohol misuse (67% of the sample reported having been drunk on alcohol). The prevalence is likely now higher and this may impact on observed associations. Academic results were a more objective measure and these also indicated an association with maternal immune activation. A further limitation of the study is the low proportion who were using cannabis, limiting generalizability to other settings where cannabis use is more common.

### Further Research

Future research on the association between maternal immune activation and psychosis could consider a broader range of maternal immune markers. In addition, it is important to consider how the associations with cannabis use and academic performance may relate to later risk for psychotic disorders. We lacked sufficient numbers to consider this question but it would be possible in a larger cohort. A larger cohort could also further explore factors influencing resilience and susceptibility to maternal immune activation that were not considered in this study. There is also considerable scope for animal model studies to further explore the neurochemical and behavioral effects of offspring substance use on maternal immune activation, integrating evidence on specific neuroreceptors and brain regions.

### Conclusions

Building on the literature suggesting links between prenatal maternal infection, immune activation, and adult schizophrenia, using a prospective birth cohort, we report that maternal immune activation is also associated with known risk factors for schizophrenia and psychotic disorders, notably adolescent academic performance and cannabis use. These findings add to our understanding of how prenatal maternal immune activation may be associated with psychotic disorders in offspring.

## Data Availability

Data are available from the Northern Finland Birth Cohort (NFBC) for researchers who meet the criteria for accessing confidential data (data accession number P0081). Please, contact NFBC project center (NFBCprojectcenter@oulu.fi) and visit the cohort website (www.oulu.fi/nfbc) for more information.
